# Understanding phage BX-1 resistance in *Vibrio alginolyticus* AP-1 and the role of quorum-sensing regulation

**DOI:** 10.1128/spectrum.02435-24

**Published:** 2025-01-14

**Authors:** Xiaoyu Li, Xin Liu, Tianyi Ma, Haochen Su, Bingrui Sui, Lili Wang, Bilal Murtaza, Yongping Xu, Na Li, Demeng Tan

**Affiliations:** 1 MOE Key Laboratory of Bio-Intelligent Manufacturing, School of Bioengineering, Dalian University of Technology, Dalian, China; 2 Department of Infectious Diseases, Zhongshan Hospital, Fudan University 12478 , Shanghai, China; 3 Shanghai Public Health Clinical Center, Fudan University, Shanghai, China; Centre de Biologie Integrative, Toulouse, France

**Keywords:** *Vibrio alginolyticus*, quorum sensing, bacteriophage, phage receptor, bacterial cellulose

## Abstract

The marine ecosystem is characterized by a rich diversity of bacterial hosts and their phages. The propagation of phages is primarily limited by their ability to adsorb to host cells and is further challenged by various bacterial defense mechanisms. To fully realize the potential of phage therapy in aquaculture, a comprehensive understanding of phage-host interactions and their regulation is essential. In this study, we isolated a novel *Myoviridae* phage, BX-1, capable of infecting *Vibrio alginolyticus* AP-1, and characterized its resistant mutants. We elucidated the essential role of the bacterial cellulose biosynthesis-related gene *bcsE*, which functions as a cyclic di-GMP-binding protein, in influencing host susceptibility to phage BX-1. Interestingly, Congo Red, Calcofluor White staining, and cellulose content assays indicated that deletion of *bcsE* in strain AP-1 does not completely abolish cellulose production, suggesting that *bcsE* is not essential for bacterial cellulose synthesis. Furthermore, investigating the signaling molecules that regulate phage-host interactions, we find that in a high cell density state (Δ*luxO*), bacterial cells upregulate their susceptibility to phage BX-1, which leads to a rapid development of resistance. Conversely, cells in a low-density state (Δ*hapR*) exhibit reduced susceptibility to phage BX-1 while still producing comparable phage progenies. This population density-dependent response is primarily enhanced by the predicted quorum-sensing autoinducer CAI-1, synthesized by the gene *cqsA*. Collectively, our findings reveal the intricate dynamics of phage-host interactions, adding a new layer of complexity to our understanding of phage receptor regulations.

**IMPORTANCE:**

Phage therapy has garnered significant attention as a promising solution to antibiotic resistance in aquaculture. However, its application is hindered by a limited understanding of the genotypic and phenotypic dynamics governing phage-host interactions. Bacteria have developed various defense mechanisms against phages, such as mutations in phage receptors. In this study, we demonstrate that the bacterial cellulose biosynthesis-related gene *bcsE* plays a crucial role in determining susceptibility to phage BX-1, while quorum-sensing (QS) systems significantly influence collective phage-related behaviors. By characterizing the mechanisms of phage resistance and the regulatory role of QS in susceptibility, our findings enhance the understanding of phage-host interactions and pave the way for more effective phage therapy applications. Collectively, these insights illuminate the evolutionary complexities of phage-defense systems and the broader strategies that bacteria employ to coexist with phages.

## INTRODUCTION

Phages, the most abundant life forms in marine ecosystems, play an important role in shaping bacterial populations, diversity, and nutrient cycles and contribute to bacterial genome evolution through their various life cycles (1). The formidable capacity of phages to lyse bacteria has reignited interest in their potential use against the causative agents of vibriosis in aquaculture (2). Research into phage therapy has been steadily growing, with specific attention given to various pathogenic *Vibrio* species, including *Vibrio alginolyticus*, *V. parahaemolyticus*, and *V. vulnificus* (3–5). These studies have revealed that lytic phages possess the capacity to effectively prevent infections and enhance the survival rates of marine aquacultures at various developmental stages. However, the swift emergence of phage resistance poses a challenge to phage therapy and restricts its potential applications. Therefore, gaining insights into the intricate relationships between phages and their hosts is crucial for efficient phage management.

Adsorption marks the initial phase during which phages adhere to the surface of bacterial cells. The primary cause of phage resistance is believed to be receptor mutations (6). However, the prevalence of such mutations in phage receptors within complex marine environments remains uncertain due to the significant fitness costs they may incur (7). While resistance mechanisms stemming from mutations operate at the unicellular level, safeguarding individual cells and their offspring, there is mounting evidence that quorum-sensing (QS) systems regulate group behaviors of bacteria, playing a crucial role in population-level phage defense strategies in response to cell density (8–12). With increasing cell density, the likelihood of phage infection is anticipated to increase. Therefore, if cells can monitor population density to adjust their phage defense, hosts may potentially alleviate the metabolic burden associated with genetic mutations, thereby conferring evolutionary advantages for maintaining host fitness. Numerous studies have demonstrated the involvement of QS systems in the interplay between phages and bacteria, encompassing phage adsorption, bacterial biofilm formation, phage lysis-lysogeny conversion, coevolution of bacteria and phage, and information exchanges within phage populations (8, 9, 11, 13–17). In *Escherichia coli*, QS suppresses the expression of the phage lambda receptor protein LamB, while in *V. anguillarum*, it inhibits the expression of outer membrane protein K (OmpK), restricting phage KVP40 infection. Similarly, in *V. cholerae*, QS downregulates O-antigen production, thereby hindering phage adsorption (8, 11, 14). Additionally, QS stimulates the expression of *cas* genes and enhances CRISPR adaptation in *Serratia* sp. and *Pseudomonas aeruginosa* (9, 18). Conversely, QS can increase the rate of phage adsorption by upregulating the expression of phage receptors such as type IV pili (T4P) and O-antigen in *P. aeruginosa* (16, 19). Thus, there are significant intra- and inter-species variations in QS-mediated phage-host regulation.

Within *V. alginolyticus*, three cognate membrane-bound receptors, LuxN, LuxPQ, and CqsS, individually facilitate the binding of specific autoinducers (AI-1, AI-2, and CAI-1) (20–22). These receptors initiate a signaling cascade that ultimately leads to the expression of the crucial transcription factor HapR, which regulates QS genesin a density-dependent manner (23). When the cell density is low, LuxO is active and suppresses the expression of *hapR*. However, when the cell density is high, LuxO is deactivated by dephosphorylation, which activates the expression of *hapR*. This allows the global regulator HapR to control gene expression (23). If LuxO is deactivated, HapR is fully expressed at all densities, leading to a locked high-cell density (HCD) phenotype. Conversely, if HapR is deactivated, it loses the ability to regulate QS-associated phenotypes, resulting in a locked low-cell density (LCD) phenotype.

Research into the interplay between phage predation and QS has primarily focused on *V. cholerae*, *V. parahaemolyticus*, and *V. anguillarum*, with limited understanding regarding *V. alginolyticus. V. alginolyticus*, a notable Gram-negative marine pathogen, poses a significant risk to the worldwide aquaculture industry, contributing to increased rates of mortality and morbidity across diverse species, including fish, shrimp, and shellfish (24). Specifically, due to antibiotic overuse, *V. alginolyticus* has now emerged as a prominent marine pathogen responsible for skin ulceration in cultured sea cucumbers (*Apostichopus japonicus*) (25). Investigating QS-mediated phage-host interactions could elucidate synergistic effects between phages and QS-signaling molecules or quenching enzymes. Hence, further exploration is urgently needed to understand the potential antimicrobial strategies stemming from such interaction, particularly regarding the impact of QS on phage defense mechanisms at the population level.

In this study, we investigated the intricate interactions between *V. alginolyticus* strain AP-1 and a novel lytic phage BX-1, focusing on the genetic determinants of phage resistance and the influence of QS on phage-host dynamics. We identified the *bcsE* gene as essential for the successful infection by phage BX-1, although it is not required for bacterial cellulose production. Notably, we found that the susceptibilities to phage BX-1 in strain AP-1 are upregulated by QS. Overall, our findings contribute to the understanding of QS-regulated phage-host interactions and underscore the complex regulatory mechanisms of phage defense in response to environmental factors.

## RESULTS

### Isolation and characterization of phage BX-1

**Fig 1:**
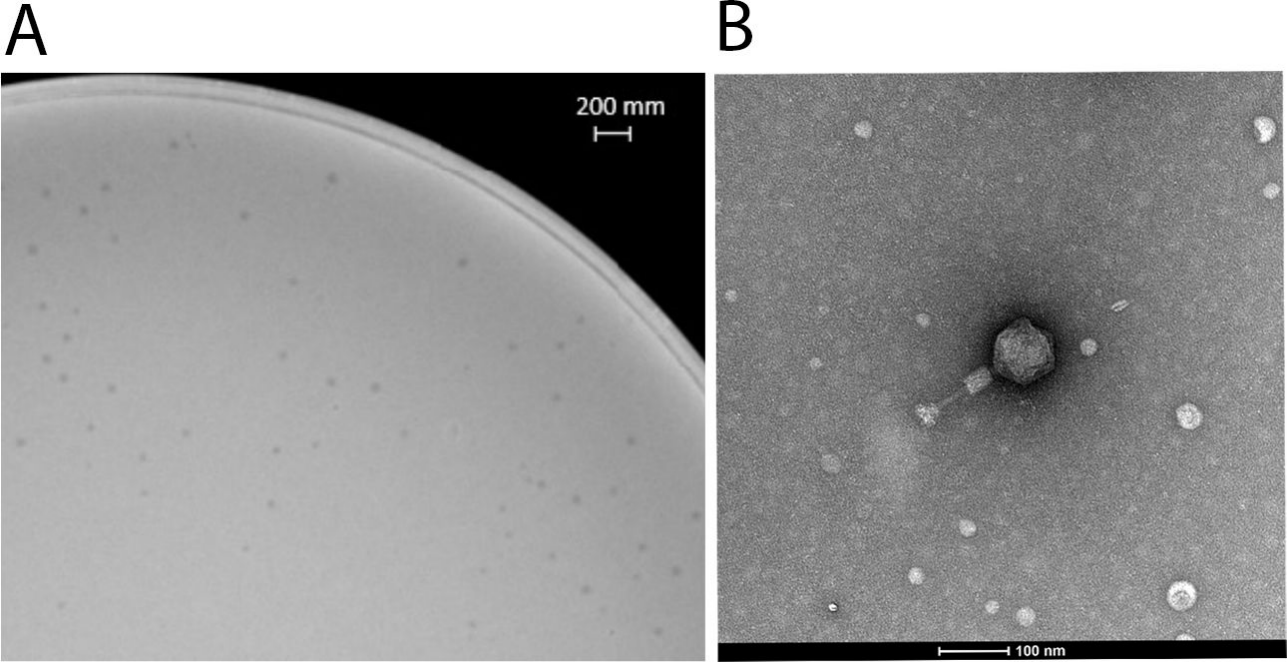
Characterization of plaque morphology and structure of phage BX-1. (**A**) Plaque morphology of phage BX-1 observed on its host strain, *V. alginolyticus* AP-1, scale bar 200 mm. (**B**) Transmission electron microscopy image of phage BX-1, with a scale bar representing 100 nm.

To isolate vibriophages, sewage samples were enriched in Luria broth (LB) medium, and the resulting cell-free supernatant (CFS) was collected. The enriched CFS was incubated with exponentially growing *V. alginolyticus* strain AP-1 for 6 h. Following incubation, the CFS was harvested, sterilized with 1% chloroform, and applied to lawns of strain AP-1. A single plaque, designated as phage BX-1, was isolated from the AP-1 lawn (Fig. 1A). Host range assays indicated that BX-1 has a narrow host range, infecting only strains AP-1 and ZVA while showing no infectivity toward other closely related environmental isolates (Fig. S1). Morphological analysis classified BX-1 within the *Myoviridae* phage family (Fig. 1B). It features an icosahedral head measuring approximately 86 nm in diameter, a contractile tail around 129 nm long, and a base portion with multiple tail fibers (Fig. 1B). Genomic sequencing revealed BX-1 to have a linear double-strand DNA genome spanning 144,654 bp, with an overall G + C content of 41.9%. The genome context of BX-1 closely resembles that of marine vibriophages such as VAP-7 (accession no: NC_048765.1, with 100% query cover and 99.7% identity) and VP-1 (accession no: MH363700.1, with 98% query cover and 98.05% identity). The genome of BX-1 does not contain any integrase, toxin, or virulence factor genes. Interestingly, an aspartate transfer RNA gene was identified (Fig. S2), which is likely to enhance phage codon usage, especially for late genes (26).

### BcsE cellulose biosynthesis protein: a necessity for phage BX-1 adsorption

**Fig 2:**
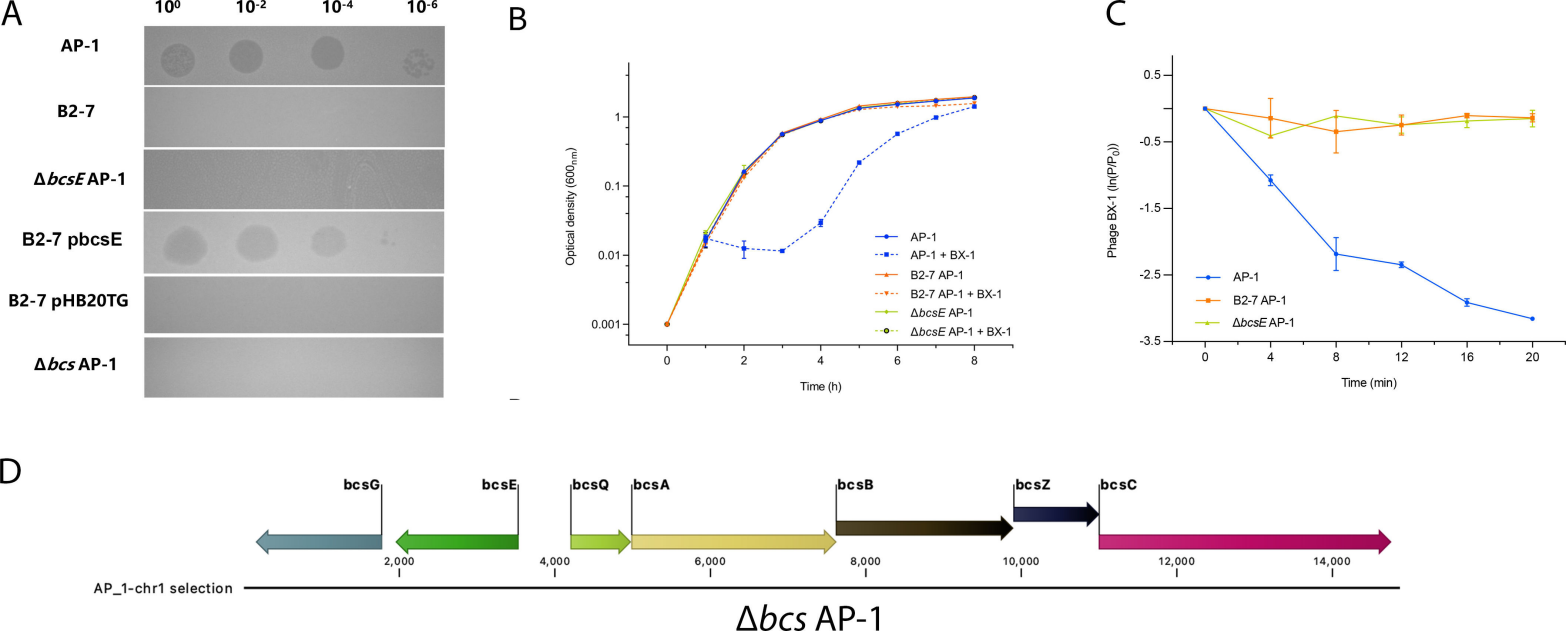
Identification and characterization of the phage BX-1 resistance gene. (**A**) A spot test was conducted using a 100-fold dilution series of phage BX-1 on the wild-type strain of *V. alginolyticus* (AP-1), along with the phage-resistant mutants B2-7, Δ*bcsE* AP-1, and B2-7 pbcsE, harboring the plasmid pHB20TG, which expresses the *bcsE* gene under the control of an arabinose-inducible promoter, B2-7 carrying an empty plasmid (pHB20TG), a mutant lacking most bacterial cellulose biosynthesis genes. The image shown is representative of three biological replicates. (**B**) The removal of the *bcsE* gene conferred complete resistance to phage BX-1 infection in a liquid medium assay, as there was no observable growth difference between the phage-resistant mutant and Δ*bcsE*, regardless of the presence or absence of phage BX-1. (**C**) Adsorption assay demonstrates that the mutation in the *bcsE* gene impairs the adsorption process of phage BX-1. At the specified time point, unadsorbed phage BX-1 was quantified using a plaque assay. (**D**) Schematic representation of the predicted gene cluster associated with bacterial cellulose biosynthesis in strain AP-1 and the cellulose-deficient mutant Δ*bcs* AP-1.

Next, we randomly picked and purified microcolonies that had survived exposure to the phage BX-1 from the spot test assay (Fig. S1) and then performed spot test to evaluate their complete resistance to BX-1 accordingly (27). To validate this, we employed comparative genomic analysis to pinpoint bacterial genes linked to phage resistance accordingly. We sequenced four resistant mutants with heritable phenotypes after three rounds of purification and spot tests. All of them carry mutations in the *bcsE* gene. One of the mutants (B2-7 AP-1) possesses a T insertion in the CDS, beginning at the 258th nucleotide position, leading to the creation of a truncated peptide of 96 amino acids (AAs) due to an early stop code. The complete BcsE protein comprises 528 AAs and functions as a cytosolic c-di-GMP-binding protein involved in bacterial cellulose biosynthesis. Notably, previous research has demonstrated that phage S6 employs bacterial cellulose as a receptor to infect *Erwinia amylovora*, the causative agent of fire blight (28). These findings strongly suggest that the gene *bcsE* may play a significant role in phage infection in *V. alginolyticus* AP-1. Subsequently, we constructed an isogenic mutant, Δ*bcsE*, and evaluated its plating efficiency and growth dynamics. Consistent with our expectations, phage BX-1 was unable to form a plaque on the Δ*bcsE* AP-1 strain or inhibit its growth (Fig. 2A and B), suggesting a direct link between the inactivation of the *bcsE* gene and resistance to phage BX-1. When complemented with the *bcsE* of the wild-type strain AP-1, the phage-resistant mutant B2-7 regained susceptibility to phage BX-1. As expected, the introduction of an empty plasmid, pHB20TG, into the resistant mutant B2-7 did not yield the same result (Fig. 2A). Collectively, these results lead us to the conclusion that gene *bcsE* plays a crucial role in phage BX-1 infection. To determine if a mutation in the *bcsE* gene affects phage BX-1’s binding ability to strain AP-1, we conducted a phage adsorption assay. This assay quantified the amount of unabsorbed phage BX-1 over time. We noted varying adsorption rates between the wild-type strain AP-1, the phage-resistant mutant B2-7, and the Δ*bcsE* AP-1 mutant (*P* < 0.001; Fig. 2C). After incubating with the host strain AP-1 for 20 min, about 96% of phage BX-1 was adsorbed (Fig. 2C). In contrast, less than 13% of phage BX-1 was adsorbed by the phage-resistant mutant B2-7 and Δ*bcsE* AP-1 within the same timeframe.

### BcsE is not essential for bacterial cellulose production

**Fig 3:**
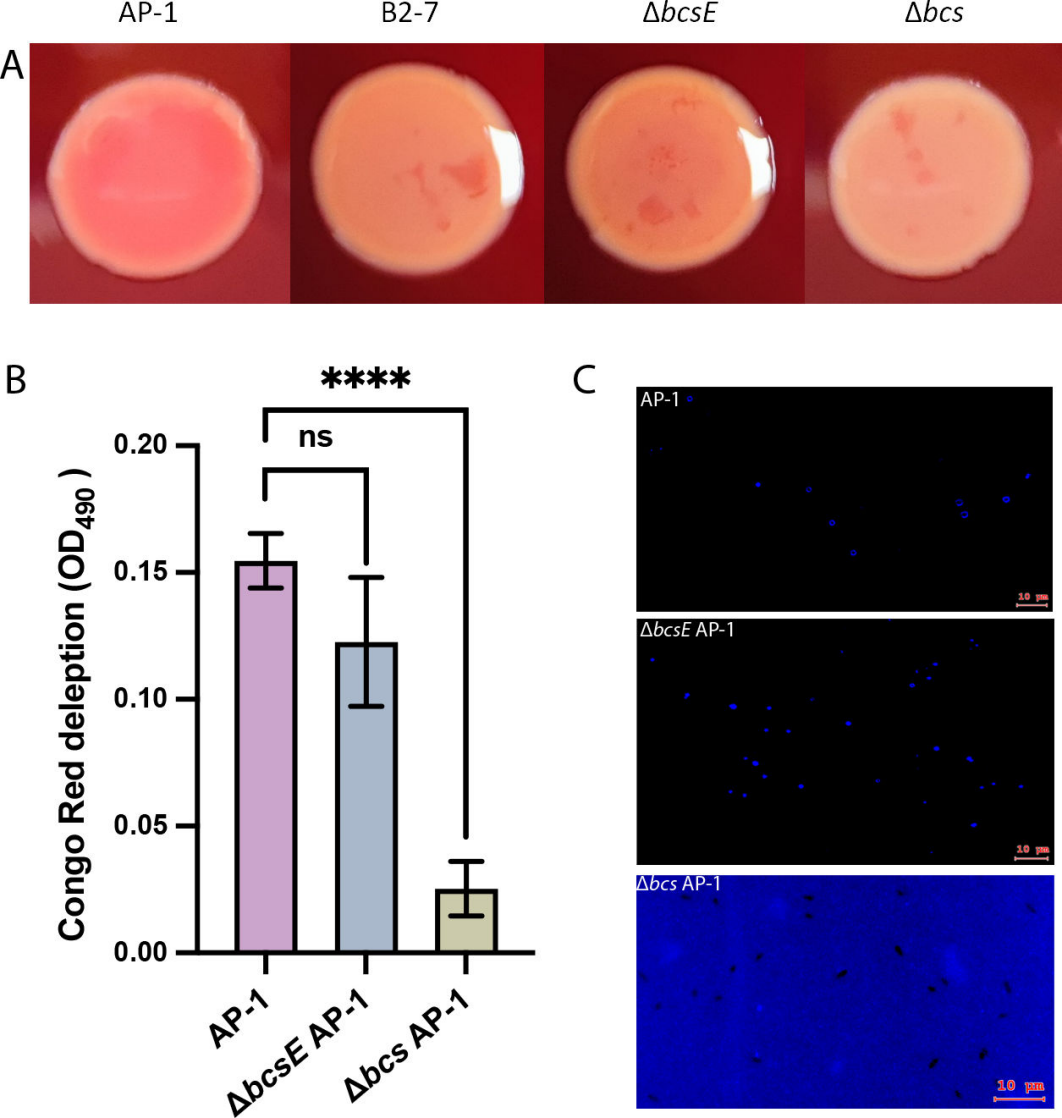
Cellulose content assay of strains AP-1 and Δ*bcsE* AP-1. (**A**) Representative images from the Congo Red assay comparing the wild-type *V. alginolyticus* strain AP-1, the phage BX-1-resistant mutant B2-7, the Δ*bcsE* AP-1 mutant, and the Δ*bcs* AP-1 mutant. (**B**) Quantification of cellulose content among the wild-type strain AP-1, the Δ*bcsE* AP-1 mutant, and the Δ*bcs* AP-1 mutant was conducted by harvesting bacterial cells during exponential growth, followed by mixing with Congo Red. The depleted Congo Red was quantified by measuring optical density (OD), accounting for the adsorption of free Congo Red in control cultures without bacterial pellets. (**C**) Representative images showing Calcofluor White binding to strains AP-1 and Δ*bcsE* AP-1 but not to the Δ*bcs* AP-1 mutant, as observed under an epifluorescence microscope. Scale bar, 10 µm. Data are presented as means ± SD from three independent biological samples. One-way analysis of variance (ANOVA) was used for multiple comparisons (****P < 0.0001; NS, not significant).

Research on *Salmonella enterica* has demonstrated that while *bcsE* is not essential for bacterial cellulose synthesis, it can significantly enhance cellulose secretion (29). To determine whether the *bcsE* gene is essential for bacterial cellulose production in *V. alginolyticus* AP-1, we employed Congo Red, a dye that binds to 1,4-β-glucose polymers, and Calcofluor White, a non-specific fluorochrome that specifically interacts with cellulose in cell walls, as indicators of cellulose production. This provides the basis for a rapid and sensitive screening test to determine the presence of cellulose (30, 31). Through the straightforward observation of Congo Red binding in bacteria cultured on agar plates, we successfully distinguished the cellulose production levels of the wild-type strain AP-1 and its derivatives, with the wild-type exhibiting a vibrant pink hue. In contrast, the phage-resistant B2-7 mutant and the Δ*bcsE* AP-1 strain displayed a more subdued yellowish tint, indicating variations in cellulose synthesis (Fig. 3A). Furthermore, we constructed a mutant, Δ*bcs* (Fig. 2D), which lacks most of the biosynthesis genes *bcsGEABZC*. This mutant exhibits resistance to phage BX-1 and displays a distinctive white appearance (Fig. 2A and 3A). This strongly supports the association among phage resistance, bacterial cellulose, and Congo Red binding ability. Notably, the deletion of the *bcsE* gene had a minor effect on the content of Congo Red in the CFS. In contrast, the deletion of the *bcsGEABZC* gene cluster significantly (*P* < 0.0001) blocked Congo Red binding in liquid assays (Fig. 3B). In accordance with the Congo Red assay, Calcofluor White staining revealed significant binding in both the AP-1 and Δ*bcsE* AP-1 strains, resulting in blue, round-shaped cell walls. In contrast, no binding was observed in the Δ*bcs* AP-1 strain (Fig. 3B; Fig. S3). These results strongly suggest that the deletion of the *bcsE* gene does not fully abolish bacterial cellulose production. Since cellulose can be hydrolyzed into β-D-glucose under acidic conditions, we measured the cellulose content in strains AP-1 and Δ*bcsE* using an anthrone chromogenic assay in the presence of strong acid. Compared to the wild-type strain AP-1, there was an approximate 15.8% reduction in cellulose content (Fig. S4). Despite the absence of the *bcsE* gene not eliminating bacterial cellulose production, we speculate that the altered spatial and temporal distribution of cellulose may still influence the ability of phage BX-1 to attach and initiate infection.

### Influence of conditioned medium on phage-host interactions

**Fig 4:**
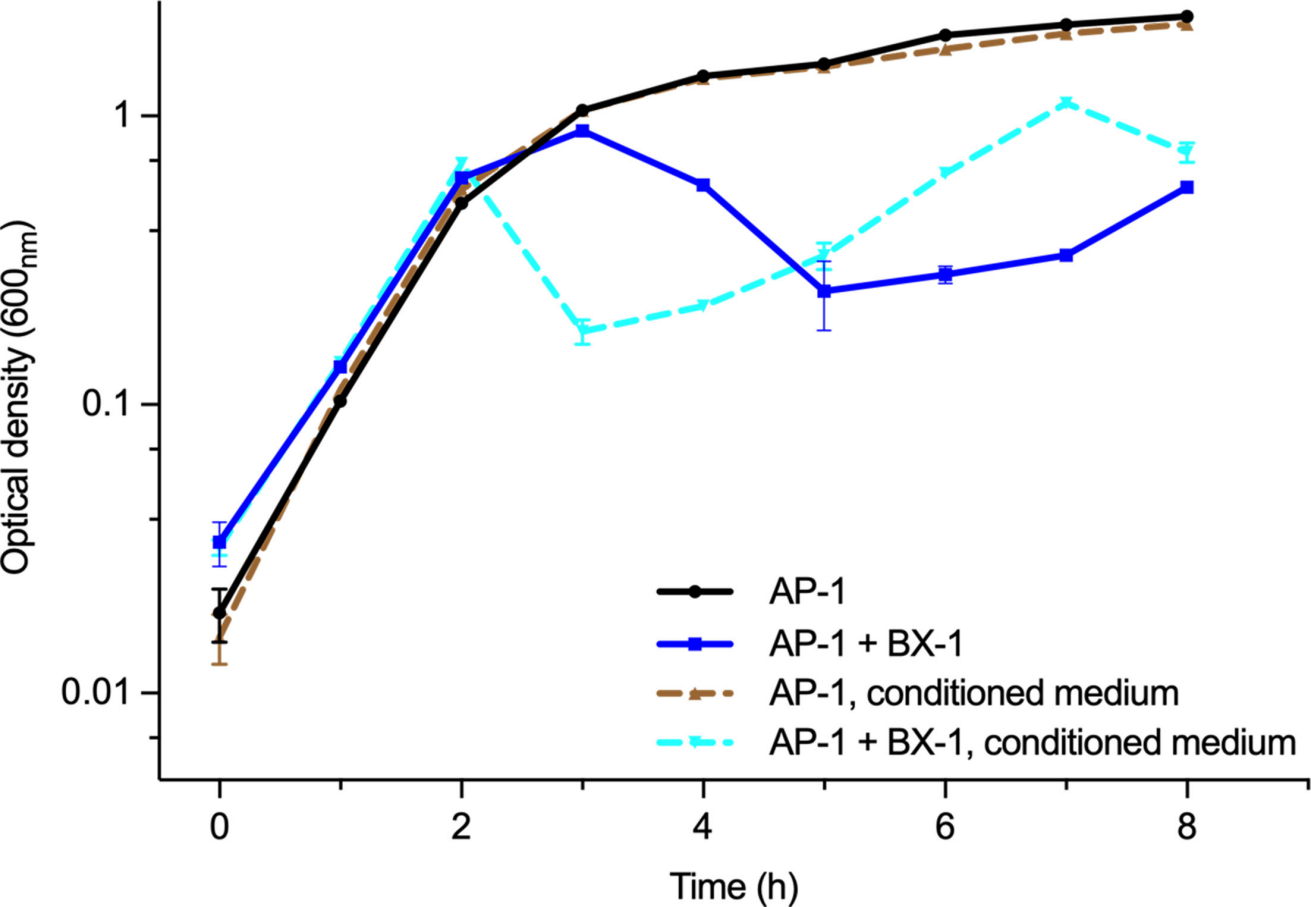
Influence of external molecules on phage-host interactions. Growth curves of *V. alginolyticus* AP-1, which was subjected to infection by phage BX-1 at a multiplicity of infection (MOI) of 1, in conditioned and LB medium, respectively. The conditioned medium used was derived from a mid-log phase culture of wild-type *V. alginolyticus* AP-1 with an OD of 1.0. Measurements of optical density at 600 nm were taken from culture samples every hour for a duration of 8 h. The data shown represent three biological replicates (*n* = 3).

In our previous study, we demonstrated that the addition of CFS to a mixture of *V. anguillarum* PF430-3 and KVP40 completely prevented the phage-induced aggregation phenotype (11). Similarly, when a mixed culture of *V. cholerae* containing both phage and bacteria was supplemented with exogenous autoinducers CAI-1 and AI-2, it led to enhanced survival of *V. cholerae* and reduced production of phages. We initiated the study to test the hypothesis that bacteria secrete signaling molecules that govern phage-host interactions (8, 11, 14, 16). For this, we prepared conditioned medium from cultures of *V. alginolyticus* isolates strain AP-1 growing at mid-log phase with an OD of 1.0, mimicking HCD status, and CFS was collected and filtrated to eliminate bacteria. Concentrated LB medium was incorporated into the conditioned medium to counteract any differences in growth rate caused by nutrient fluctuations. The growth rate remained unaffected in conditioned mediums, with strain AP-1 reaching a stationary phase after 3 h of incubation (Fig. 4). Interestingly, the infection dynamics of phage BX-1 in the conditioned medium of AP-1, as deduced from the bacterial growth, differed from those in the fresh LB medium. In the conditioned medium, strain AP-1 underwent lysis 2 h post phage BX-1 infection, while in the control LB medium, the culture showed more resistance to lysis, commencing only 3 h after the addition of phage BX-1. These observations imply that cell density-dependent regulation, influenced by external factors such as autoinducers, could potentially expedite the lysis of strains AP-1 by phage BX-1.

### QS-mediated the host susceptibilities of phage BX-1

**Fig 5:**
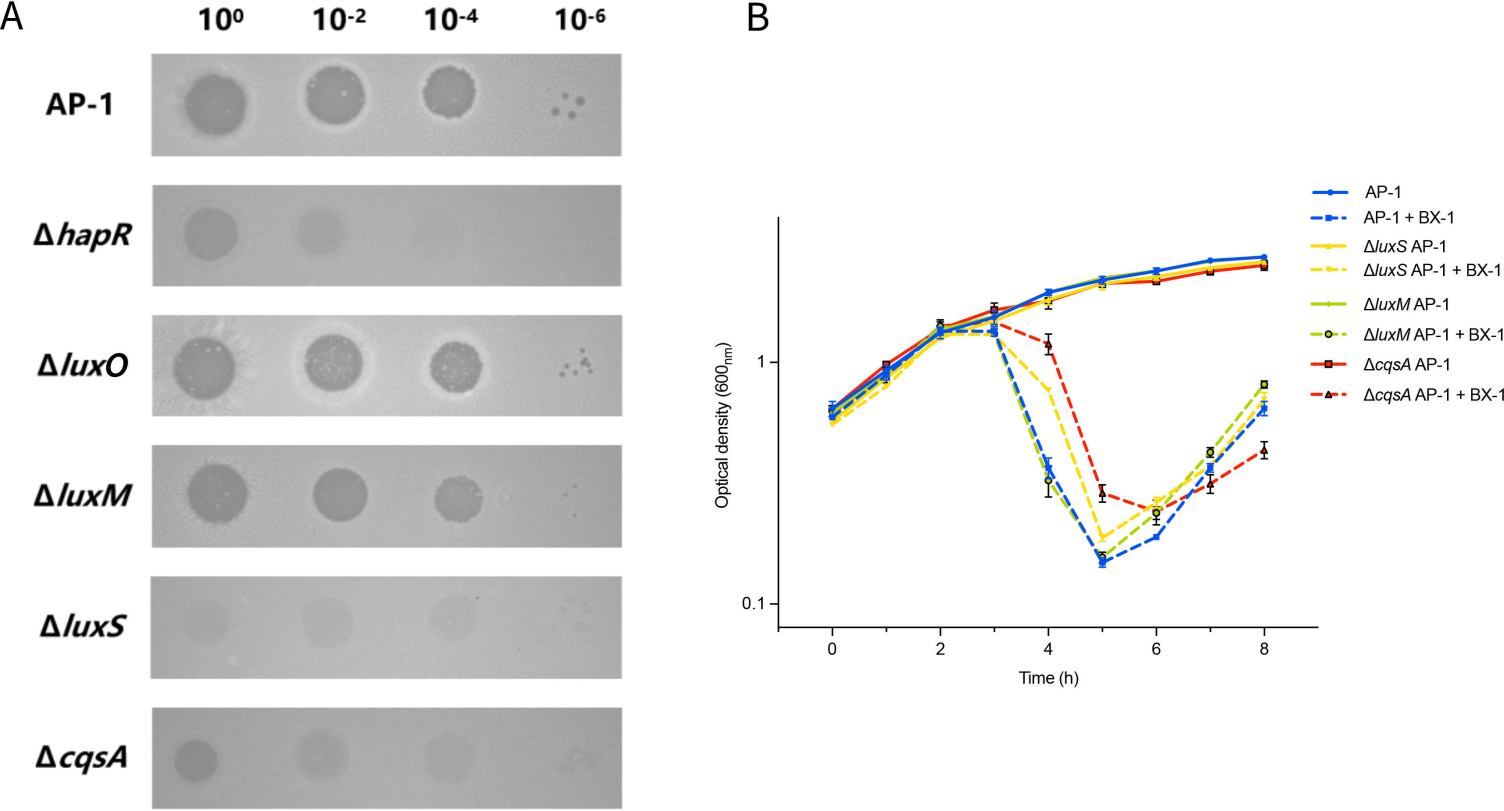
Inactivation of QS autoinducer synthases influences phage-host interactions. (**A**) Spot test assay on *V. alginolyticus* strains AP-1 and its QS derivatives using phage BX-1. The phages were spotted in 100-fold serial dilutions on the bacterial lawns. The Δ*hapR* AP-1 mutant showed a decrease in susceptibility to phage BX-1 and a change in plaque morphology from transparent to turbid, compared with the wild-type strains AP-1 and Δ*luxO* AP-1. The QS synthase mutants Δ*cqsA* AP-1 and Δ*luxS* AP-1 also showed decreased susceptibility to phage BX-1, while behaving similarly to the Δ*hapR* AP-1 mutant. Images are representative of three biological replicates. (**B**) Growth curves of *V. alginolyticus* strains AP-1 and QS synthase mutants in the presence and absence of phage BX-1 in LB medium. Data represent an average of three biological replicates (*n* = 3).

To test the hypothesis that bacteria secrete signaling molecules that influence phage-host interactions, potentially including V. alginolyticus, we investigated how QS impacts the interactions between phage BX-1 and its V. alginolyticus host strains. We first assessed the optical density and hapR mRNA levels in the AP-1 strain, and our results revealed a positive correlation between optical density and hapR expression during the exponential growth phase, prior to the stationary phase (AP-1, R² = 0.709; Fig. S5). Thus, regarding the regulation of *hapR* mRNA levels, the QS circuit of *V. alignolyticus* strain AP-1 appears to operate similarly to the canonical circuit. To further explore the impact of QS on the interplay between phage and host, we selectively knocked out of the genes responsible for autoinducer (AI) synthesis, specifically *luxM*, along with the genes *luxS* and *cqsA*, which are associated with AI-1, AI-2, and CAI-1, respectively. This was done to evaluate the impact of AIs on the susceptibility of the host to phage BX-1, relative to the wild-type strain AP-1 under conditions of HCD cultures. As a result, both Δ*cqsA* AP-1 and Δ*luxS* AP-1 mutants exhibited reduced susceptibility to phage BX-1 and delayed lysis compared to wild-type strain AP-1 and Δ*luxM* AP-1, as evidenced by both the efficiency of plating (EOP) and growth inhibition assays (Fig. 5A and B).

**Fig 6:**
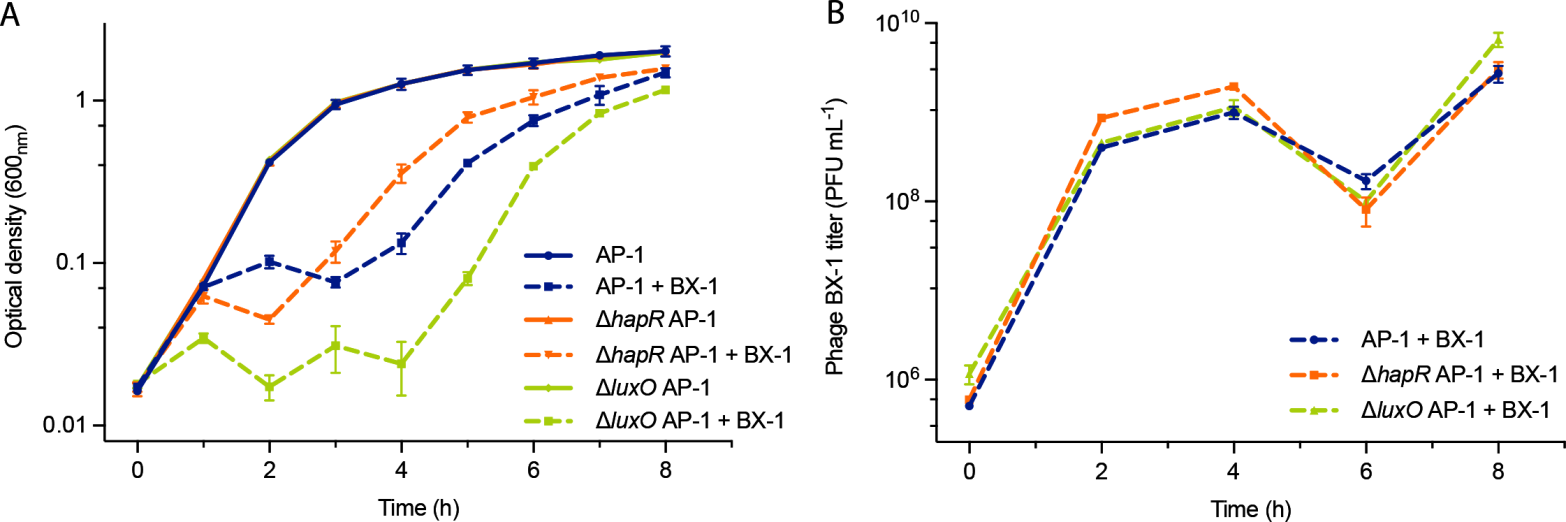
Examination of phage-host interactions in LB medium. (**A**) Optical densities (OD600) were recorded at 1 h intervals over an 8 h incubation period for strains AP-1 and the QS mutants (Δ*hapR* and Δ*luxO*), with or without phage BX-1 at MOIs of 1. (**B**) The concentration of phage BX-1 plaque forming units per milliliter (PFU mL-1) was determined at 2 h intervals over an 8 h incubation period for strain AP-1 and their QS mutants in the presence of phage BX-1. The error bars represent the SD from all duplicate experiments (*n* = 3).

Next, we created two isogenic QS mutants (Δ*hapR* and Δ*luxO*) of strain AP-1 that represent cell behavior locked at LCD and HCD irrespective of the cell culture densities. The growth kinetics of wild-type and QS mutants were evaluated by measuring the optical densities and phage concentrations. In the absence of phage infection, the growth of the AP-1 strain followed a standard population growth curve (Fig. 6A). However, the infection dynamics of QS mutants with phage BX-1 were different from those of the wild-type strains (Fig. 6A). Notably, the strain maintained in the HCD state (Δ*luxO*) displayed heightened susceptibility to phage BX-1 infection. A significant portion of the infected bacterial culture lysed within the initial 4 h, followed by the emergence of a phage-resistant subpopulation. Conversely, the wild-type strain AP-1 and the Δ*hapR* AP-1 mutant exhibited greater resistance to lysis (Fig. 6A), with regrowth occurring after 2 and 3 h, respectively. Furthermore, the count of phage BX-1 plaques over an 8 h infection period suggested that phage production was not influenced by QS in the first 6 h, but at the end, more phage was yielded in the Δ*luxO* culture (Fig. 6B). It is worth noting that at the 6 h mark, we observed a decline in phage concentration, followed by a subsequent peak, reaching its highest level over the course of the entire assay. We hypothesized that a significant portion of free-living phages may have adsorbed to the uninfected population, initiating another round of cell lysis.

Next, we assessed the infectivity of phage BX-1 on the wild-type strain AP-1, Δ*hapR* AP-1, and Δ*luxO* AP-1 by evaluating the EOPs. In contrast to the wild-type AP-1 and Δ*luxO* AP-1, the Δ*hapR* AP-1 demonstrated a lower susceptibility to phage BX-1 infection, and this was accompanied by a noticeable shift in plaque morphology from clear to turbid (Fig. 5A). Our observation contrasts with recent reports on phages KVP40 and JSF35 affecting *V. angullarum* and *V. cholerae*. In these cases, cells become resistant when they are locked in an HCD state (11, 14). In conclusion, these results further substantiate that QS influenced the susceptibilities of strain AP-1 to phage BX-1.

### QS regulates the expression of phage resistance-related gene *bcsE*

**Fig 7:**
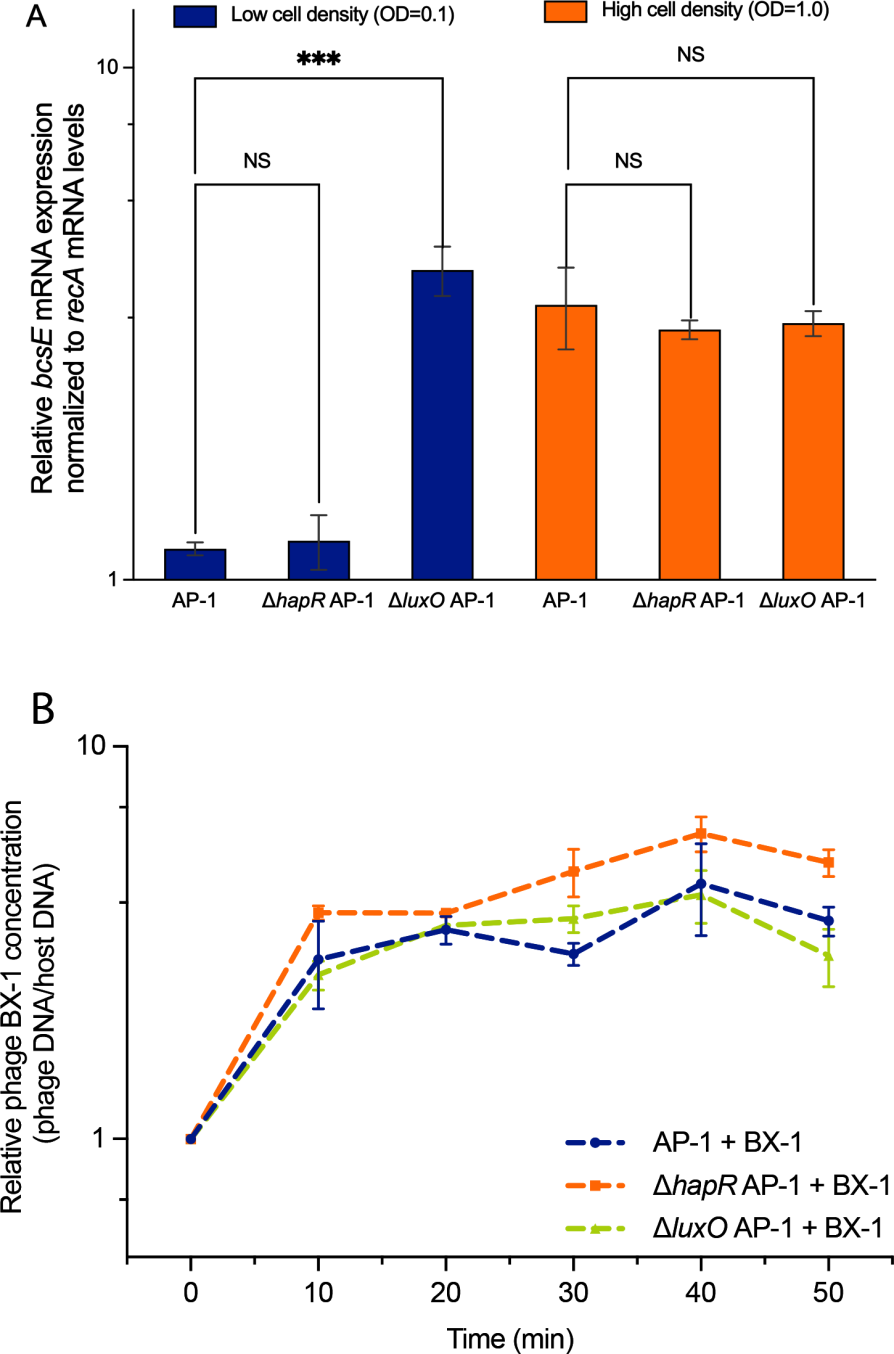
Effect of QS on the expression of the phage receptor gene *bcsE* and the replication of phage DNA. (**A**) Expression of the *bcsE* gene in the strains AP-1 and their QS mutants (Δ*hapR* and Δ*luxO*) at both low (OD = 0.1) and high (OD = 1.0) cell density cultures. (**B**) Infection of strain AP-1 and their QS mutants by phage BX-1 by using time-resolved quantitative PCR (qPCR) quantification of phage DNA copies from the intracellular fraction over the infection period. A gene coding for the major capsid protein of phage BX-1 and the housekeeping gene *recA* of *V. alginolyticus* were used to determine the relative phage genome replication. The experiments were conducted in triplicate. One-way ANOVA was used for multiple comparisons (***, *P* < 0.001; NS, not significant).

To test the frequency of bacteria resistant to phage BX-1 within the surviving AP-1 and QS mutants’ populations, a high frequency of mutations was observed in Δ*luxO* (~62.5%), in comparison to the AP-1 strain (~29.17%) and Δ*hapR* (~11.46%; *P*＜0.05; Fig. S6). These results collectively indicate a correlation between increased cell lysis and a higher mutation rate when the cells were kept under conditions akin to HCD. We hypothesized that this heightened lysis could be attributed to increased adsorption. Our earlier studies showed that the QS transcriptional factor VanT inhibited the expression of the phage KVP40 receptor *ompK* in *V. anguillarum* (11). To determine whether QS regulates genes associated with phage resistance in *V. alginolyticus*, we analyzed the expression of the *bcsE* gene in wild-type strains and QS mutants harvested from low- and high-cell density cultures. As shown, the expression of *bcsE* in strain Δ*luxO* AP-1 was roughly four times greater (*P*＜0.001) than in the wild-type strain AP-1 and Δ*hapR* AP-1 (Fig. 7A), suggesting an upsurge in the expression of the resistance gene when cells are in a regulatory state akin to HCD. These findings collectively demonstrate a link between QS signaling and the expression of resistance genes in LCD cultures. Surprisingly, in HCD cultures, the *bcsE* expression was consistent among wild-type and QS mutants, irrespective of whether cells were in a state resembling LCD or HCD (*P*＞0.05). Likewise, the growth curves indicated that strain AP-1 locked in either LCD or HCD statuses showed no difference in susceptibility to phage BX-1 infection compared to the susceptibility of the wild-type strain AP-1 in HCD inoculating cultures (OD600 = 1.0; *P*＞0.05; Fig. S7), suggesting that other environmental and metabolic signals beyond QS might potentially play a role in upregulating the adsorption process at the HCD status (32–34). For example, studies on *V. cholerae* indicated that at HCD, HapR activates the expression of *nspS-mbA*. This leads to an elevated intracellular concentration of c-di-GMP, which in turn enhances biofilm formation synergistically with AI-2 (35). It should also be noted that various gene clusters responsible for encoding proteins involved in bacterial cellulose biogenesis were not comprehensively examined. Our focus was specifically on quantifying the expression of the *bcsE* gene, while the differential expression of other related genes was not investigated. Consequently, one possible interpretation of these findings is that *bcsE* alone may not sufficiently represent the complexity of phage adsorption. In addition, one limitation of this assay is the uncertainty regarding whether the reference gene *recA* is consistently transcribed and remains unaffected by internal or external factors, including QS, in strain AP-1.

### QS and intraspecies variations affect phage BX-1 DNA replication

To explore the influence of QS on the interactions between phages and their hosts, we studied the replication process of phages BX-1 in wild-type strains and QS mutants. We collected total DNA at various time intervals following infection with phage BX-1. The amount of phage DNA within the host cells during infection was measured using quantitative real-time PCR (qPCR). In the AP-1 strain, phage BX-1 replication was marginally more efficient in the Δ*hapR* AP-1 strain, but not statically significant (*P*＞0.05), with average replication rates being 1.43 and 1.53 times higher than those of the wild-type AP-1 strain and the Δ*luxO* AP-1 strain, respectively (Fig. 7B). We propose that increased phage DNA replication in the Δ*hapR* AP-1 mutant may explain similar levels of phage production were achieved during the liquid assay, despite the reduced phage susceptibility observed simultaneously. These results support the phage production assay conducted in a liquid medium, indicating that QS does not appear to influence phage production during the early stages of infection.

## DISCUSSION

Due to the increasing antibiotic resistance in aquaculture, there is an urgent need to explore alternatives for preventing and treating bacterial infections like vibriosis. Phage therapy has garnered significant interest due to its specificity and safety. However, challenges remain as phage therapy faces obstacles such as the rapid development of resistance mechanisms, limiting its widespread potential application. In light of recent discoveries that bacteria employ various mechanisms to control phage resistance, we investigated the role of QS in managing phage-host interactions in the marine pathogen *V. alginolyticus*. This study aimed to enhance our understanding of both common and potentially species-specific traits involved in the long-term evolution of phage susceptibility regulation. Supporting this, we conducted experiments using autoinducer synthetase mutants and further investigated phage susceptibility in their respective QS-off (∆*hapR*) and QS-on (∆*luxO*) mutants. Specifically, the interaction between phage BX-1 and strains AP-1 showed that phage susceptibility increased in the Δ*luxO* strain and decreased in the Δ*hapR* strain compared to the wild-type strain. Therefore, at the population level, QS regulation of strain susceptibility may influence the outcome of interspecific phage-host interactions. Together, these findings highlight the potential synergistic roles the surrounding microbiome may play in maintaining the stability of phage and bacterial populations in diverse environments, facilitating long-term coexistence and co-evolutionary persistence.

Quantifying the bacterial cellulose-related gene *bcsE* provided additional insights into the mechanisms underlying QS-mediated phage susceptibility. However, we lack direct evidence to confirm our hypothesis that phage BX-1 uses bacterial cellulose as its receptor, as the phage-resistant mutant Δ*bcsE* still produces bacterial cellulose. We hypothesize that a mutation in the *bcsE* gene may alter the spatial and temporal distribution of cellulose, thereby significantly affecting the phage adsorption process. This hypothesis is supported by findings from Congo Red and Calcofluor White assays. In connection, studies in *E. coli* indicate that BcsE, along with BcsF, may act as accessory proteins, potentially playing regulatory roles in the installation of pEtN by BcsG (36). These findings underscore the diverse and multifaceted functions attributed to the gene *bcsE*. In the LCD cultures, the upregulation in *bcsE* expression in the Δ*luxO* strain provided direct evidence for QS-mediated upregulation of *bcsE*-mediated susceptibility. Strikingly, we found that in HCD cultures, QS had little effect on the outcome of phage BX-1-host AP-1 interactions. This was evident in the following ways (i) the susceptibilities of wild-type AP-1 and QS mutants to phage BX-1 were hardly different, and (ii) the relative expression of the phage receptor-related gene *bcsE* of wild-type and QS mutants was comparable. These findings are intriguing as they indicate that additional regulatory circuits may have been integrated into the expression of phage receptors to increase the range of environmental and metabolic signals beyond that of QS. For instance, it was demonstrated that the biosynthesis and translocation of bacterial cellulose occur in the presence of an elevated cytosolic concentration of cyclic-di-GMP (32). In *V. cholerae*, AI-2 and polyamine norspermidine increase biofilm biomass and biofilm cell density synergistically, while individually, these two molecules drive opposite biofilm formation behaviors (35). Collectively, these studies suggest that QS and c-di-GMP may work together at HCD to stimulate the production of bacterial cellulose. However, it is not known if *V. alginolyticus* strains used in this study also respond differently to these two signals. Additional research is required to elucidate the mechanisms by which bacteria coordinate QS and c-di-GMP signaling to regulate bacterial cellulose production in *V. alginolyticus*. Although studies in *E. coli* have demonstrated that specific c-di-GMP levels enhance phage N4 infectivity and can be safeguarded by phosphodiesterase PdeL, the comprehensive understanding of this integration remains incomplete (37, 38).

While QS is often seen as a beneficial mechanism that enables bacteria to adapt to challenging new environments, the seeming contradiction between QS-regulated phage adsorption and previously reported suppression of phage receptors (OmpK and O-antigen) can be explained by the multifunctional roles of phage receptors in various biological processes (14, 39). It is plausible that modification or mutation of phage receptor may impose a trade-off with fitness. For instance, we speculated that the upregulation of OmpK in LCD status in *V. anguillarum* PF430-3 may be an adaptation to stimulate extracellular nutrient uptake to adjust rapidly to changes in intracellular metabolism and environmental cues, as fully integrated outer membrane proteins are essential for the biological functions (40). However, the functionalities and persistence of OmpK have not yet been studied and remain characterized in *Vibrio* spp. For *V. alginolyticus*, biofilm formation is critical for the proper initial colonization stage and is increasingly recognized as a passive virulence factor facilitating many infectious disease processes (41). Bacterial cellulose, known as extracellular polymer, serves as a molecular catalyst to facilitate its exploration and inhabitation of diverse ecological niches and contributes to biofilm formation (42, 43). As a remarkable fibrous structural component of biofilm, it has been proposed to form a mechanically strong hydrogel with high water adsorption capacities (44). However, its role as an additional determinant for biofilm formation in *enterobacteria* is not clear. For example, Gualdi et al. showed that cellulose biosynthesis repressed curli-mediated surface adhesion and cell aggregation in the laboratory strain *E. coli*, indicating that cellulose may function as a negative determinant for biofilm formation (45). One possible explanation is that phage BX-1 infection supports both commensal and pathogenic lifestyles by degrading biofilm matrix components and disrupting its structure, thereby facilitating dissemination. This is like the activation of protease at HCD to promote the release of cells from aggregates, switching from a sessile lifestyle into planktonic cells (46). Meanwhile, increased biofilm formation can be facilitated by cellular debris and extracellular DNA (eDNA) released through phage-mediated cell lysis. In short-term growth experiments, the Δ*bcsE* AP-1 mutants grew at the same rate as the wild-type strain AP-1 in LB medium, indicating that mutations of gene *bcsE* did not affect bacterial growth at least in LB medium. Recent research on the gene *bcsE* reveals a complex array of functions. Crystallographic and functional analyses have uncovered unexpected domain architecture in BcsE, suggesting its involvement at various stages in bacterial cellulose production (47). It is proposed to influence processes ranging from early secretion system assembly to the regulation of a membrane-proximal pool of dimeric c-di-GMP for activating processive synthases (47). Although *Vibrio* spp. are prevalent in aquatic environments, research conducted on the saline waters of the Skagerrak Sea has shown that the total abundance of *Vibrio* spp ranges from 4 × 10^3^ to 9.6 × 10^4^ cells/L (48). These densities are likely associated with the LCD phenotype observed in this study. Consequently, under such circumstances, cells employ QS to suppress phage susceptibility, thereby avoiding phage attacks dependent on phage receptor. Thus, examining the interplay between QS and potential phage receptors, along with other defense mechanisms, could reveal that the HapR-regulation can vary significantly among *Vibrio* populations. This variation could influence the ecological and evolutionary roles in the long-term relationship between phage and host.

From an ecological perspective, bacteria may adopt various defense strategies depending on cell density and surrounding microbial populations to synchronize group behaviors and act collectively. Our research underscores the role of QS in the regulation of phage-host interactions at the population level, highlighting the intricate nature of antagonistic coevolution and coexistence relationships. For example, QS-upregulated susceptibility that is effective in the context of strain AP-1 may not perform as well, or could even be detrimental, in different ecological scenarios involving receptor-phage-host interactions. The mechanism by which strain AP-1 upregulates susceptibility to phage BX-1 without affecting subsequent phage BX-1 production remains unclear and warrants further investigation. Finally, within the scope of phage therapy, the rapid development of resistance must be considered for the repeated or prolonged use of phages in treatment, emphasizing that the susceptibility of *V. alginolyticus* hosts to phages is a dynamic property. This adds yet another layer of complexity to QS regulation in *V. alginolyticus*, suggesting the potential use of phage with QS molecules to increase receptor expression. This is particularly evident in *P. aeruginosa*, where the QS inhibitor Baicalin demonstrates a remarkable ability to modulate QS-controlled phenotypes. By binding to the QS transcriptional regulators LasR and RhlR, Baicalin effectively reduces their affinity for the promoter regions of target genes, showcasing its potential as a valuable tool for influencing bacterial communication and behavior (49). This inhibition may have significant implications for repressing virulence and influencing phage-host interactions. This is further supported by the fact that QS inhibitors have been shown to limit the evolution of CRISPR immunity during phage therapy (13). Additionally, T7 phage displaying quorum-quenching enzymes has inhibited biofilm formation (50). Collectively, these studies suggest that combining QS and phage therapy could be a promising strategy for future antimicrobial therapy.

## MATERIALS AND METHODS

### Bacterial strains and plasmid

Bacterial strains and plasmids used in this study are listed in Table S1. *V. alginolyticus* strain AP-1 was routinely grown at 37°C in LB medium or on 1.5% agar plates aerobically. Antibiotics were added at the following concentrations: 25 µg mL^−1^ chloramphenicol and 15 µg mL^−1^ gentamycin for *E. coli* and 5 µg mL^−1^ chloramphenicol and 15 µg mL^−1^ gentamycin for *V. alginolyticus*.

### Phage BX-1 isolation and purification

Phages were obtained from wastewater runoff samples collected at the aquatic products section of a local supermarket, using *V. alginolyticus* AP-1 as the host strain. The isolation and purification of phages followed previously established procedures (11). A single translucent phage plaque on the plate was selected, and after three purification steps, phage BX-1 was obtained. The propagation of phage BX-1 was carried out using the double-layer plaque assay standard method (51). Phage lysate was stored at 4°C until further use.

### Examination of phage BX-1 morphology

An aliquot of phage BX-1 lysate (~1 × 10^7^ PFU mL⁻¹) was applied as a drop onto 200-mesh carbon-supported copper grids and allowed to incubate at room temperature for 10 min. Subsequently, the sample was negatively stained with 2% (wt/vol) phosphotungstic acid for 3 min, followed by the removal of excess liquid using filter paper. The phage particles were then visualized using a TEM HT7800 microscope (Hitachi, Tokyo, Japan). The images of phages were analyzed and quantified using ImageJ software (https://imagej.net/ij/).

### Genomic DNA extraction and sequencing

The phage BX-1 genome was extracted using DNA Purification with the DNeasy Blood and Tissue Kit accordingly (52). The extracted phage genome DNA was then sent to Shanghai Human Genome Center for sequencing. Subsequently, Newbler software was utilized for data assembly, resulting in the acquisition of the phage genome sequence. Gene function prediction was conducted using GeneMarks (http://topaz.gatech.edu/GeneMark/genemarks.cgi) and RAST (http://rast.nmpdr.org/rast.cgi). The clusters of orthologous groups (COG) classification was carried out using the CDD database. Additionally, the functional annotation mapping of the phage genome was conducted using the Proksee online platform (https://proksee.ca/).

### Bacterial genome manipulation

To construct chromosomal deletion in *V. alginolyticus* strains AP-1, the flanking regions of the target genes were amplified with the primers listed in Table S2 accordingly (53). The first PCR products, which contain ~30 bp overlap of identical sequence, were used as templates for a second PCR to splice DNA fragments together. The PCR product was digested with restriction enzymes and cloned into the corresponding sites of plasmid pDM4. Bacterial conjugation was carried out between the recipient strains and donor strains at a ratio of 10:1. Briefly, overnight bacterial cultures of strain *V. alginolyticus* and *E. coli* S17-1 harboring the suicide plasmid were 100-fold diluted in LB medium and incubated at 37°C to reach OD ~ 1.0. The mixture was spotted onto an LB agar plate and incubated at 37°C overnight. Transconjugants were selected on thiosulfate-citrate-bile salts-sucrose (TCBS) agar plates (Oxoid, Hampshire, United Kingdom) supplemented with chloramphenicol (5 µg mL^−1^) (54). Individual transconjugants were picked and purified on LB agar plate, followed by selection for plasmid loss on LB agar containing 10% sucrose at room temperature for 48 h. Colonies sensitive to chloramphenicol were selected for PCR verification and subsequently Sanger sequenced.

The *bcsE* gene was amplified from the wild-type strain *V. alginolyticus* strain AP-1 using primers listed in Table S2. The resulting PCR products were digested with EcoRI and XbaI FastDigest Restriction Enzymes (Thermo Fisher Scientific, CA, USA) and ligated (T4 DNA Ligase, Thermo Fisher Scientific, CA, USA) into the plasmid pHB20TG. The resulting plasmid (designated as pHB20TG_bcsE) was transferred into the *E. coli* component cell DH5α and subsequently verified by PCR and Sanger sequencing. The constructed plasmid was then transferred into the phage BX-1-resistant mutant *V. alginolyticus* strain B2-7 by conjugation and selection of gentamycin-resistant colonies on TCBS agar plates. The expression of *bcsE* was induced by the addition of 0.4% L-arabinose followed by spot tests.

### Comparative genomic analysis of phage BX-1 resistance gene

Phage-resistant colonies were successfully isolated from a spot test assay conducted with phage BX-1 and strain AP-1. Randomly selected microcolonies were restreaked and subsequently assessed for their resistance using the spot test (55). The DNA of phage-resistant mutants was extracted using the Wizard Genomic DNA Purification Kit from Promega, following the manufacturer’s instructions. The quality of the extracted DNA samples was checked using a nanodrop and agarose gel electrophoresis. The bacterial genomes were then sent to Sangon Biotech (Shanghai, China) for Illumina sequencing. Assembled bacterial genomes were aligned to the reference wildtype strain *V. alginolyticus* AP-1 reference genome for further analysis as previously described (27).

### Phage adsorption assay

To investigate the impact of alterations in the *bcsE* gene on phage adsorption, overnight bacterial cultures were diluted 1,000-fold in LB medium and grown to an OD of 0.3 at 37°C. Phage BX-1 was introduced at an MOI of 0.001. Samples were collected at 4, 8, 12, 16, and 20 min post-infection. To obtain cell-free supernatant, the cell suspension was centrifuged at 16,000 × *g* for 2 min at 4°C. Unabsorbed phage particles were quantified by plaque assay with the host strain AP-1. The reduction in unabsorbed phage was calculated using the formula y = (ln[P0] − ln[*P*])/B0, where P_0_ and B_0_ represent the initial numbers of phages and bacteria, respectively, at *t* = 0, as previously described (15). This analysis was based on the average of three independent experiments.

### Characterization and quantification of bacterial cellulose

For the Congo Red agar assay, an aliquot of 2 µL from overnight bacterial cultures was spotted onto plates supplemented with Congo Red, achieving a final concentration of 4 mg mL⁻¹, and incubated at room temperature for 48 h. Cellulose production, associated with the *bcs* genes, was indicated by the appearance of pink colonies, while gene-disrupted mutants exhibited a white phenotype. For the liquid assay, overnight bacterial cultures were diluted 1,000-fold in 2 mL of LB medium. Cells were harvested at an OD of 0.4 at 8,000 × *g* for 3 min at 4°C. The bacterial pellet was resuspended in 2 mL of phosphate-buffered saline (PBS) buffer, and Congo Red was added to a final concentration of 160 µg mL⁻¹. The suspension was incubated statically for 6 h. The CFS was collected, and the residual Congo Red concentration was measured by recording the OD at 490 nm. The depletion of Congo Red was determined by subtracting the absorbance measurements of each individual sample from the absorbance of the control PBS buffer containing Congo Red without bacterial addition.

Next, cellulose content was determined using a detection kit (BC4280, Solarbio LifeSciences) in accordance with the manufacturer’s instructions utilizing a cellulose colorimetric method (56–58). Briefly, overnight bacterial cultures of strains AP-1 and Δ*bcsE* AP-1 were diluted to an OD of 0.01 and cultivated to the exponential phase. Once the cultures reached an OD of 0.4, the cells were harvested. Concentrated sulfuric acid was then added to hydrolyze the bacterial cellulose into β-D-glucose under acidic conditions. The cellulose content was quantified using a carbazole-H_2_SO_4_ colorimetric method, with absorbance measured at 620 nm using a visible spectrophotometer.

For the Calcofluor White staining procedure, overnight cultures of strains AP-1, Δ*bcsE* AP-1, and Δ*bcs* AP-1 were harvested via centrifugation at 8,000 × *g* for 3 min and resuspended in PBS. Calcofluor White Stain (Merck, Cat. No. 18909–100ML-F) was added to the bacterial suspensions in a 1:1 ratio and incubated at room temperature for 30 min, following the manufacturer’s protocol. Subsequently, 2.5 µL aliquots were mixed with preheated agarose gel, transferred onto glass slides, and visualized using epifluorescence microscopies (IX-83, Olympus and TissueFAXS 200, TissueGnostics GmbH), respectively.

### Phage infection assay of *V. alginolyticus* wild-type AP-1 and its derivative mutants

The susceptibility of *V. alginolyticus* wild-type AP-1 and its QS mutants to phage BX-1 was evaluated by the EOP. Briefly, overnight bacterial cultures were back diluted and grown to the exponential phase. Aliquots of 200 µL of bacterial culture were mixed with 4 mL of melted top agar and allowed to solidify at room temperature. Serially diluted phage stocks were spotted on the bacterial lawn and incubated overnight at 37°C. The lytic potential of phages BX-1 was tested in LB medium and parallel control without phages. Briefly, overnight culture was diluted to average ODs of 0.017, and a 10 µL aliquot of phage lysate (up to 1.35 × 10^10^ PFU mL^−1^) was added at average MOIs of 0.7 to strain AP-1 and its QS mutants, along with fresh LB broth in parallel. Phage concentration was regularly quantified using plaque assays. The initial determination at 0 h was made after the phage had been added and cultured for 10 min.

### Real-time quantitative PCR assay

Overnight bacterial cultures of *V. alginoluticus* strains were back diluted 10,000-fold in LB broth and grow at 37°C. Aliquots were harvested for RNA extraction for *bcsE* mRNA quantification from the inhibition experiments above. Total RNA was extracted using the TRIzol method according to the manufacturer’s protocol (Thermo Fisher Scientific). Residual genomic DNA was removed by DNase I treatment and subsequently inactivated by EDTA at 80°C for 5 min. DNA-free RNA was stored at −80°C until further analysis. cDNA was obtained using the Thermo Scientific Revert Aid First-Strand cDNA Synthesis Kit as described by the manufacturer protocol with 0.5 µg RNA template (Thermo Fisher Scientific). For real-time PCR, relative expression levels of gene *bcsE* relative to *recA* mRNA were determined using SsoAdvanced SYBR Green Supermix (Bio-Rad) as described previously (59).

Phage infection time courses for relative abundance were performed at an MOI of 4 accordingly (60, 61). Bacterial pellets were harvested and washed three times in LB medium to remove unabsorbed phages, followed by total DNA extraction using DNeasy Blood and Tissue Kits (QIAGEN). Aliquots of 5 µL diluted template DNA (0.2 ng) were mixed with SYBR green qPCR mix (SsoAdvanced Universal SYBR Green Supermix, Bio-Rad) with the following program: 30 s at 95°C for denaturation followed by 40 cycles of 4 s at 95°C and 30 s at 55°C. The relative quantification of the phage target genes (phage major capsid protein) to the *V. alginolyticus recA* was determined using the comparative threshold cycle (CT) method outlined above (59). At each time point, the relative phage concentration in each individual strain was normalized by the relative replication number calculated using the comparative threshold at *t* = 0.

### Statistical analysis

Statistical analysis and graphs were generated using GraphPad Prism (https://www.graphpad.com/). All values are expressed as their mean ± SD. Levels of significance were evaluated using one-way ANOVA for multiple comparisons or Student’s *t*-test for comparison between the two groups. *P < 0.05, **P < 0.01, ***P < 0.001; and ****P < 0.0001; NS, not significant.

## Data Availability

The sequence data of bacteria and phage genomes, AP-1 (ASM1693762v1) and BX-1 (OK428602.1), are available in GenBank. The data of the sources utilized to generate all main and Fig. S1 in this study are accessible in Supplementary data.

## SUPPLEMENTAL MATERIAL

Supplemental data spectrum.02435-24-s0001.xlsx The data of the sources utilized to generate all main and supplementary figures.

Supplemental material spectrum.02435-24-s0002.docx Tables S1 and S2; Fig. S1 to S7.

ASM does not own the copyrights to Supplemental Material that may be linked to, or accessed through, an article. The authors have granted ASM a non-exclusive, world-wide license to publish the Supplemental Material files. Please contact the corresponding author directly for reuse.
