# Supplementary material for: Understanding phage BX-1 resistance in Vibrio alginolyticus AP-1 and the role of quorum-sensing regulation
Source: Microbiol Spectr. 2025 Jan 14;13(2):e02435-24. doi: 10.1128/spectrum.02435-24 (PMC11792527; doi:10.1128/spectrum.02435-24)
Supplement: Supplemental material — Tables S1 and S2; Fig. S1 to S7. [file spectrum.02435-24-s0002.docx]

Table S1. Bacterial strains, plasmids and bacteriophage

| Name | Genotype or relevant markers |
| --- | --- |
| *Escherichia coli* |  |
| S17-1 | *thi* pro *hsdR* *hsdM*+*recA* RP4-2-Tc::Mu-Km::Tn7 λ*pir* |
| DH5α | In this study |
| *Vibrio alginolyticus* |  |
| AP-1 | *V. alginolyticus,* isolated from sea cucumber |
| Δ*bcsE* AP-1 | In-frame deletion of *bcsE* for AP-1 |
| Δ*bcs* AP-1 | In-frame deletion of *bcs* for AP-1 |
| Δ*hapR* AP-1 | In-frame deletion of *hapR* for AP-1 |
| Δ*luxO* AP-1 | In-frame deletion of *luxO* for AP-1 |
| Δ*luxM* AP-1 | In-frame deletion of *luxM* for AP-1 |
| Δ*luxS* AP-1 | In-frame deletion of *luxS* for AP-1 |
| Δ*cqsA* AP-1 | In-frame deletion of *cqsA* for AP-1 |
| ZVA | *V. alginolyticus,* isolated from turbot |
| V3  V7  V9  V11 | In this study  In this study  In this study  In this study |
| pDM4 | Cm^R^; suicide vector with an R6K origin (pir requiring) and *sacB* genes from *Bacillus subtilis* |
| pHB20TG | This study |
| pbcsE | Gm^R^; pHB20TG derivative containing *bcsE* |
| pΔbcs | Cm^R^; pDM4 derivative deleting *bcs* in-frame of AP-1 |
| pΔbcsE | Cm^R^; pDM4 derivative deleting *bcsE* in-frame of AP-1 |
| pΔhapR | Cm^R^; pDM4 derivative deleting *hapR* in-frame of AP-1 |
| pΔluxO | Cm^R^; pDM4 derivative deleting *luxO* in-frame of AP-1 |
| pΔluxM | Cm^R^; pDM4 derivative deleting *luxM* in-frame of AP-1 |
| pΔluxS | Cm^R^; pDM4 derivative deleting *luxS* in-frame of AP-1 |
| pΔcqsA | Cm^R^; pDM4 derivative deleting *cqsA* in-frame of AP-1 |

Table S2. Oligonucleotides used in this study

| Name | Primers (5'-3') |
| --- | --- |
| *bcsE* deletion | |
| bcsE_1 | CCGCTTGAGCCCAAAGACGG |
| bcsE_2 | GATTTAAGGCTCTTTATGAATATTAACGATTTTTTACTGACCGTG |
| bcsE_3 | ATCGTTAATATTCATAAAGAGCCTTAAATCAATAACTTAATGTGTTTTGTG |
| bcsE_4 | TTTCTCGAGCCCGCCACTATCATTTTCAG |
| bcsE_5 | GACACATTGCAGGTTTGGCG |
| bcsE_6 | GCTGACGGCCATACGCGGAC |
| bcsE_7 | GCAATCCGAGTATTGCTTGG |
| bcsE_8  *bcs* deletion  bcs_1  bcs_2  bcs_3  bcs_4  bcs_5  bcs_6 | GGCTTTGAAGACTTGCCAC  TTTTCTAGAGAGAACGCGCTCCATCATG  CCGGCCTTTGTTTTTTGCGAGAGTAAAACCATGCAATGACAAAGG  ATGGTTTTACTCTCGCAAAAAACAAAGGCCGGAAATCCCGGCCTTTGTTG  AATAGCCAAGCTCGAGCTGC  CGACAGCGTCAATAACCACG  GCCCAAGAACTACTGAACGC |
| *hapR* deletion | |
| hapR_1 | TTTTCTAGAGGCATCAAGTACGAAGTAGC |
| hapR_2 | GGCAAGGAAAATGGATAGCTTAGCTCGTAATATATGTCAGCCCAC |
| hapR_3 | TATTACGAGCTAAGCTATCCATTTTCCTTGCCATTTGAGTTGATATTG |
| hapR_4 | TTTCTCGAGGCACGTGCCAAGTCTGCCAT |
| hapR_5 | GTACACTGAGCCAGAAGTGG |
| hapR_6 | GCCGTAGCTTGACGCAGTC |
| *luxO* deletion | |
| luxO_1 | TTTTCTAGACTACGAGCGCAGTTCATCG |
| luxO_2 | ATACATTTACACTCATAACCTAGCTATTATTTTGTTGTGTTTTAAACAAT |
| luxO_3 | AAAATAATAGCTAGGTTATGAGTGTAAATGTATTAAATCAGCAAAAAATA |
| luxO_4 | CGATAAGCATCTCGAGTTTC |
| luxO_5 | CCGTCGTCGCGAGAAGCAGG |
| luxO_6 | CGAGACTGGCGTCACGATAG |
| *luxM* deletion | |
| luxM_1 | TTTTCTAGAGCTAGTGCGACAAAGACGAC |
| luxM_2 | GAGCTAGGTTATATCATGCTTGATGTTCATTTGCACGGTCTCTTTTACCC |
| luxM_3 | ATGAACATCAAGCATGATATAACCTAGCTCTAACGTTTTGTTATATTTTG |
| luxM_4 | TTTCTCGAGGGTCAAGCGGGTGTTCCTGG |
| luxM_5 | CTGGCTTGGACCACTAATGG |
| luxM_6 | GGCATGGGCGCTTACATTGC |
| *luxS* deletion | |
| luxS_1 | TTTTCTAGACTCATGAAACACGGCGTCAG |
| luxS_2 | CTCTTAATGAGCACTACATCTCTCCTGATTGTAACTCCAGTACCTTTCGG |
| luxS_3 | CAATCAGGAGAGATGTAGTGCTCATTAAGAGAAGCATATTGCCCCAATAAG |
| luxS_4 | TTTCTCGAGGAAGGCAGCACCAATATTCG |
| luxS_5 | GGTCAGGGTGGCTCTCGTAC |
| luxS_6 | GTACGGTGATATCATCGGTC |
| *cqsA* deletion | |
| cqsA_1 | TTTTCTAGACTACTCATGTTTCAAGGCGC |
| cqsA_2 | CTACACACTAACAATAAATCTCTTCTTCATATGGAAAAACGCCAGC |
| cqsA_3 | GAAGAAGAGATTTATTGTTAGTGTGTAGATAAAACTAAAGAGCTCAACAG |
| cqsA_4 | TTTCTCGAGCTGATGGATATCGAAATGCC |
| cqsA_5 | GAAGTCGAGATTGAGACCGC |
| cqsA_6 | GAAAGAAGACGGGCAGCAGG |
| qPCR | |
| recA_F | ACCGCATTTATTCAACCACC |
| recA_R | GCATACCCCACTTTTCAGAC |
| bcsE_F | CATTTCCCACTCAACACACTC |
| bcsE_R | ACAACGTCGCCATCTCTTC |
| hapR_F | AAAGTATGGTTCGAGTGGAGTG |
| hapR_R | GCAACTGGTTAGTACGGTTAGT |
| BX-1_F | GAGTACCCAAGACGCTGAAA |
| BX-1_R | GCGAATAGACCACGAGACTTAG |


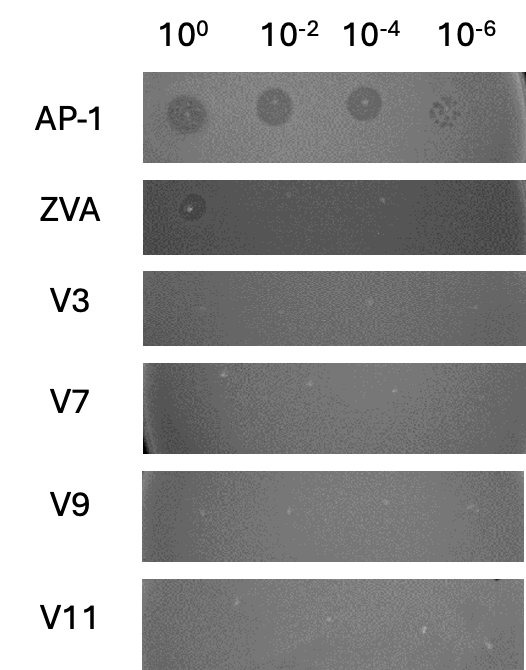


Figure S1. Spot test of phage BX-1 against *Vibrio alginolyticus* isolates collected in this study. Phage BX-1 only successfully infected strains AP-1 and ZVA, demonstrating a narrow host range.


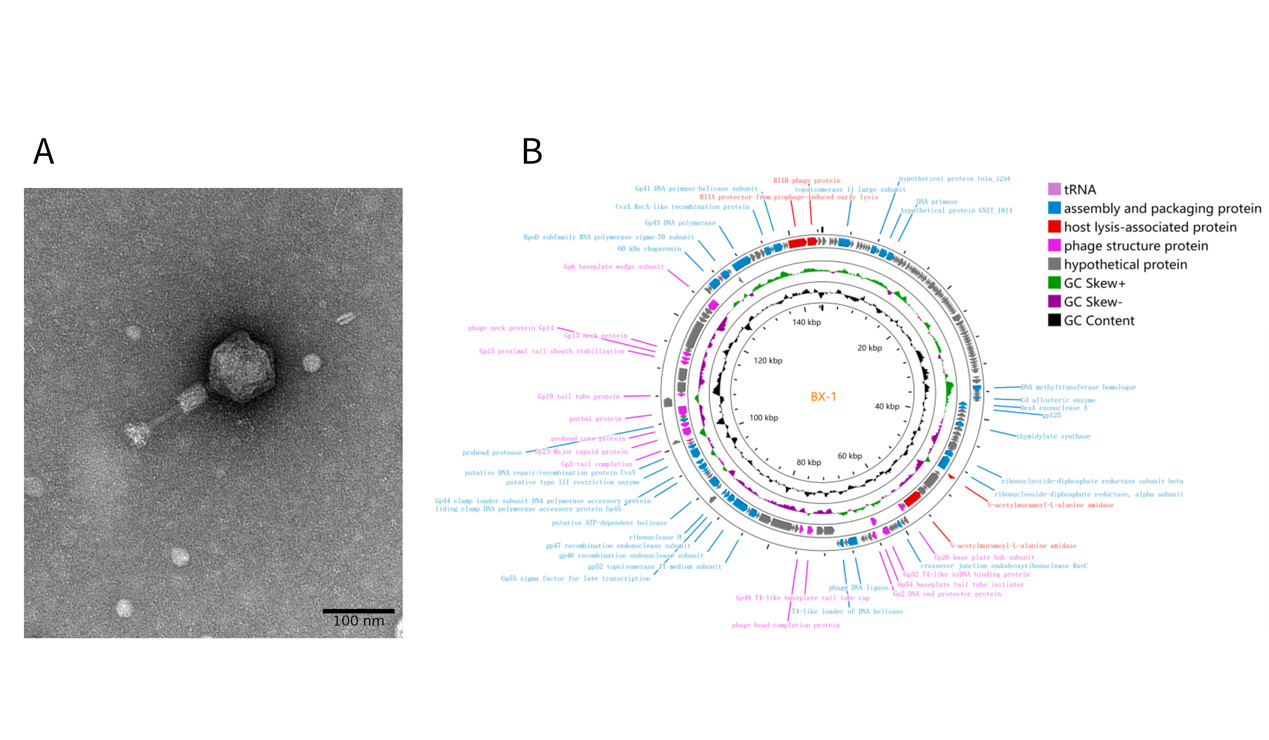


Figure S2. Schematic representation of the genomic characteristics of phage BX-1.


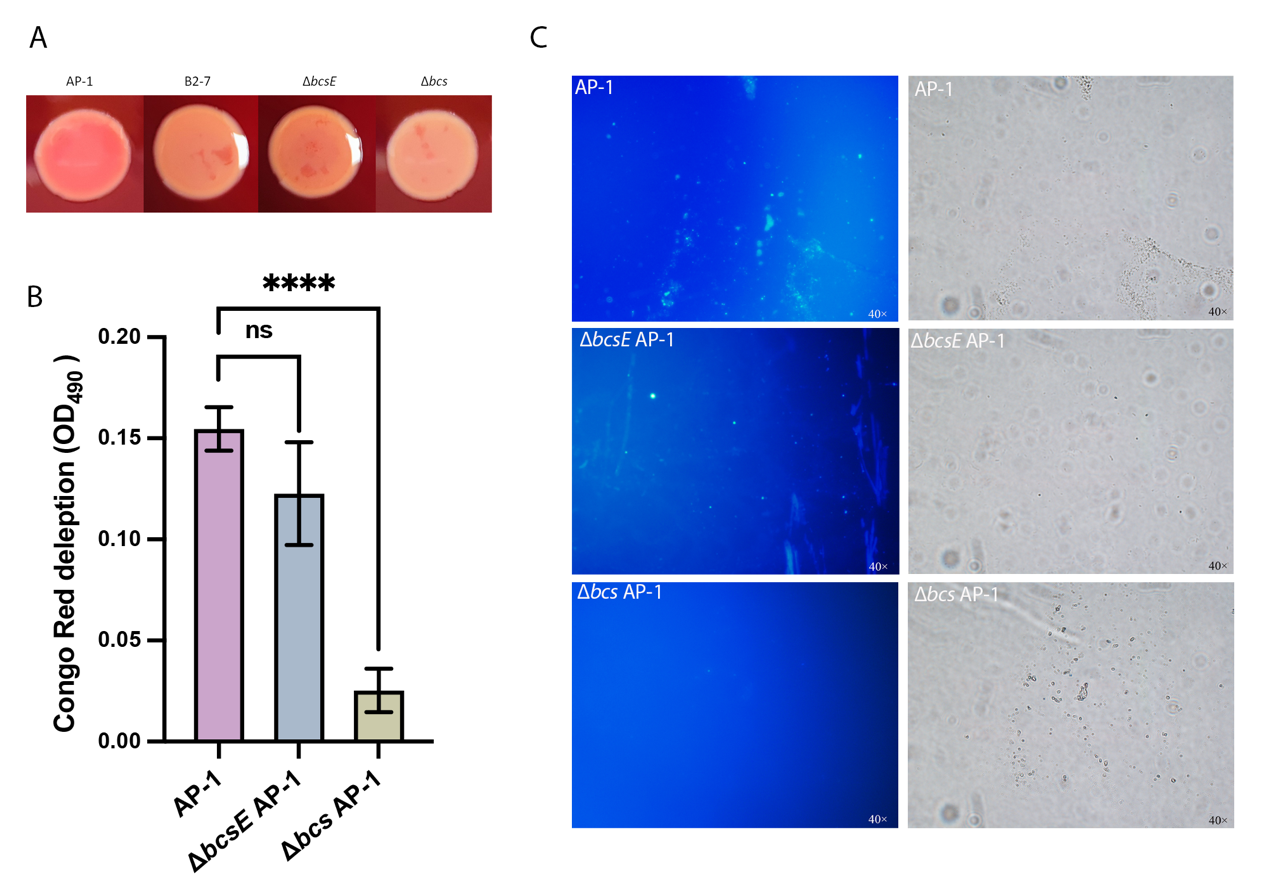


Figure S3. Representative images of Calcofluor White binding assays for the wild-type strain AP-1, the Δ*bcsE* AP-1 mutant, and the Δ*bcs* AP-1 mutant, captured using phase-contrast and fluorescence microscopy. Scale bar, 10 µm.

Figure S4. Cellulose content was quantified in strains AP-1 and Δ*bcsE* AP-1 by harvesting bacterial cells during exponential growth and normalizing the content to optical density (OD). Values are presented as the mean and standard deviation from three independent biological samples. One-way ANOVA was performed for multiple comparisons (**, P < 0.01; NS, not significant).

Figure S5. The correlation between optical density (OD600nm) and *hapR* expression in the wild-type strain AP-1. Aliquots of cultures were harvested over the incubation for measurement of optical density and gene expression by RT-qPCR.


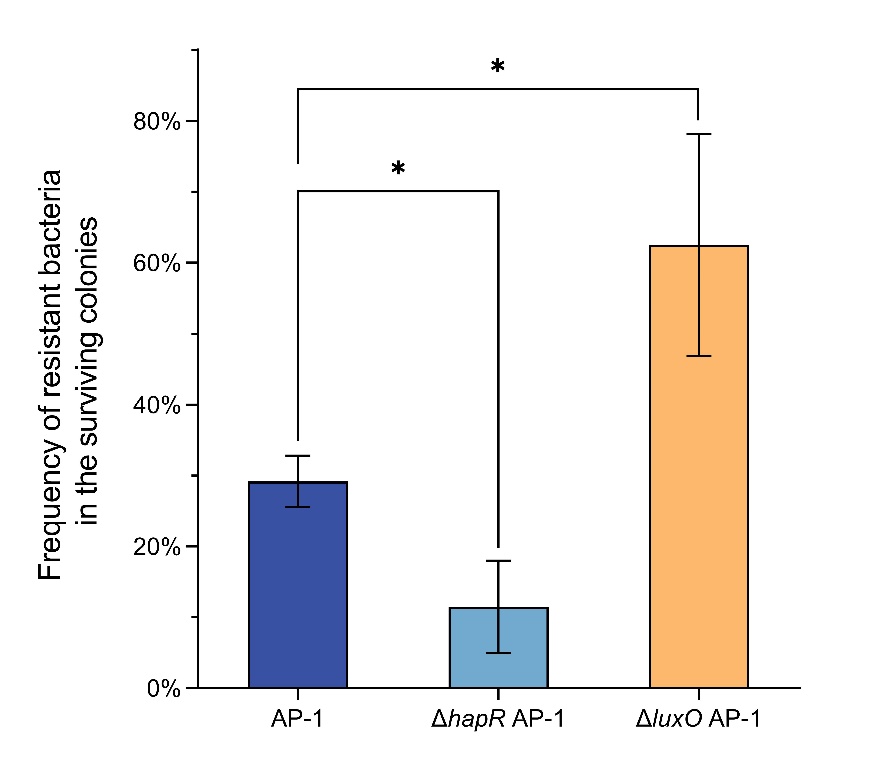


Figure S6. Frequencies of bacteria fully resistant to phage BX-1 were assessed from colonies surviving the phage infection. Up to 96 microcolonies from each strain (AP-1, Δ*hapR* AP-1, and Δ*luxO* AP-1) were restreaked onto fresh plates to generate "offspring" colonies from individual surviving cells. Randomly selected offspring colonies from each microcolony were subsequently tested for resistance to BX-1 via spot tests. Experiments were conducted in triplicate. *, P < 0.05 (paired t test).


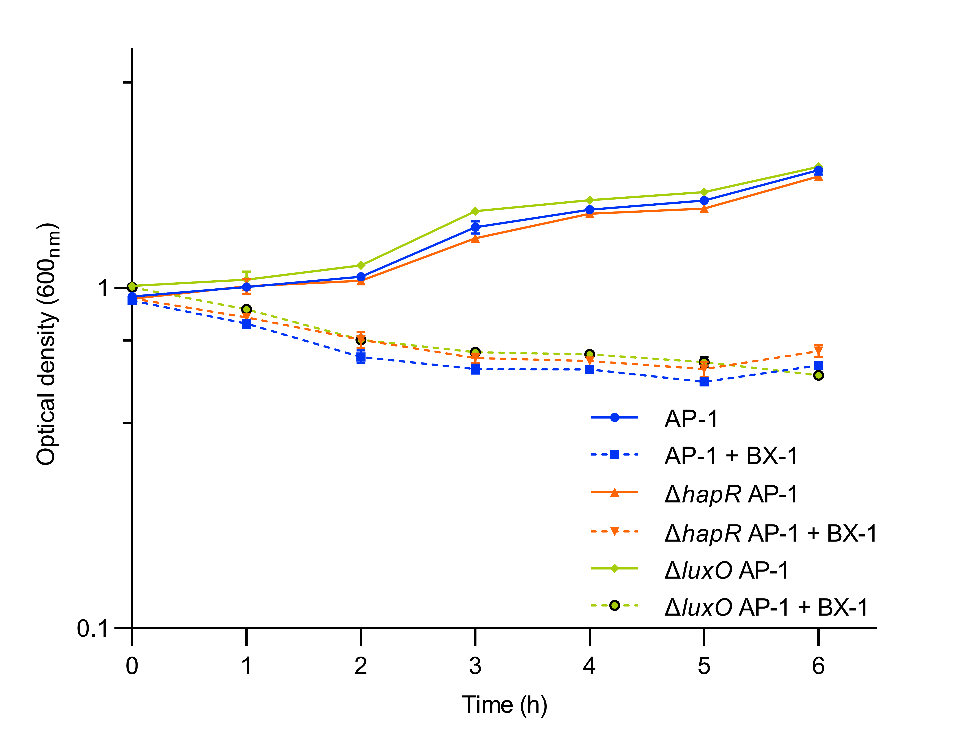


Figure S7. Growth curve of *V. alginolyticus* AP-1 and its QS mutants in the absence or presence of phage BX-1. Overnight bacterial cultures were 1000-fold back diluted and grown to an OD of 1.0 and infected with phage BX-1 at MOIs of 1. Optical densities of bacterial cultures were measured at 1-h intervals over a 6-h period of incubation.
